# Genetic diversity and population structure of *Quercus fabri* Hance in China revealed by genotyping‐by‐sequencing

**DOI:** 10.1002/ece3.6598

**Published:** 2020-07-24

**Authors:** Shifa Xiong, Yunxiao Zhao, Yicun Chen, Ming Gao, Liwen Wu, Yangdong Wang

**Affiliations:** ^1^ State Key Laboratory of Tree Genetics and Breeding Chinese Academy of Forestry Beijing China; ^2^ Research Institute of Subtropical Forestry Chinese Academy of Forestry Hangzhou China

**Keywords:** China, genotyping‐by‐sequencing, genetic diversity, population structure, *Quercus fabri*

## Abstract

Analysis of genetic diversity and population structure among *Quercus fabri* populations is essential for the conservation and utilization of *Q. fabri* resources. Here, the genetic diversity and structure of 158 individuals from 13 natural populations of *Quercus fabri* in China were analyzed using genotyping‐by‐sequencing (GBS). A total of 459,564 high‐quality single nucleotide polymorphisms (SNPs) were obtained after filtration for subsequent analysis. Genetic structure analysis revealed that these individuals can be clustered into two groups and the structure can be explained mainly by the geographic barrier, showed gene introgression from coastal to inland areas and high mountains could significantly hinder the mutual introgression of genes. Genetic diversity analysis indicated that the individual differences within groups are greater than the differences between the two groups. These results will help us better understand the genetic backgrounds of *Q. fabri*.

## Graphical abstract

The analysis of genetic diversity and population structure among white oak populations is essential for the conservation and utilization of white oak resources. We present a diversity study of a collection of 158 Q. Fabri individuals from 13 representative natural distribution areas in China using genotyping‐by‐sequencing (GBS). We found that the genetic structure was explained mainly by their geographic location. The genetic structure of adjacent populations was more similar, and infiltration of genetic background from coastal areas to inland areas was observed, where the hindrance of genetic infiltration by mountainous areas was obvious.

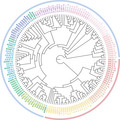

## 1 INTRODUCTION

Oak trees (Fagaceae) originated in China during the Eocene period (Aldrich & Cavender‐Bares, 2011). *Quercus* is the genus with the most species and the widest distribution in oak trees, which is found in Asia, Europe, North America, and northern Africa. *Quercus* plants in China are distributed all over the country, many of which are dominant tree species of zonal vegetation in subtropical evergreen broad‐leaved forests and warm temperate deciduous broad‐leaved forests in China, and they play an important role in China's forest ecosystem.

*Quercus fabri* Hance is one of the most ecologically and economically valuable tree species in *Quercus* in China, and it is found throughout the subtropical provinces of China (Li, Jaganathan, Zhong, Liu, & Yu, 2018). The wood of *Q. fabri* has a texture and high hardness, and furniture and flooring made of *Q. fabri* exhibit the characteristics of abrasion resistance and heavy pressure resistance (Blanchet, Beauregard, & Belleville, 2008). After processing, the fruit of *Q. fabri* is edible and it is also a source of food for many wild animals. *Q. fabri* is of great significance for water conservation and ecological protection of the Yangtze River. In addition, *Q. fabri* is also one of the important members of the colorful leaf tree species commonly used in the urban landscape layout of China.

Because of the wide geographical distribution, the complicated habitat conditions, and the ubiquitous gene exchange between species, *Q. fabri* shows a high degree of complexity in terms of genetic structure and genetic diversity. Genetic diversity analysis is the basis for plant breeding and genetic improvement, and although *Q. fabri* is an important economic tree species, its genetic diversity and population structure are still unclear. Carrying out research on the genetic diversity of *Q. fabri* resources can help to understand the current status of *Q. fabri* biodiversity and its formation and evolution history, which is of great significance for the protection of *Q. fabri* germplasm resources in China.

Berg and Hamriek (1993) and Mattila, Pakkanen, Vakkari, and Raisio (1994) have studied the genetic diversity of *Q. laevis* and *Q. robur* populations, respectively. Kim, Lee, and Hyun (1993) studied 28 populations of six oak trees including *Q. acutissima*, *Q. aliena*, *Q. dentata*, *Q. mongolica*, *Q. serrate,* and *Q. variabilis*. Gomory, Yakovlev, Zhelev, Jarmila, and Paule (2001) analyzed 25 *Q. petraea* and *Q. acutissima* populations. Chung et al. (2002) analyzed the population of *Q. acutissima*. Cavender‐Bares and Pahlich (2009) analyzed the populations of *Q. virginiana* and *Q. geminata*. Craft and Ashley (2006) conducted genetic differentiation studies on three white oak populations in northeastern Illinois. The results of these studies indicate that for the same species of oak, the genetic diversity between populations is lower, which within the population is higher, and there is a certain correlation between the genetic diversity and distribution range, population size, interspecific hybridization ability, and habitat conditions; and the genetic diversity of different oak is very different, which is caused by the difference in plant life history and evolution history (Berg & Hamriek, 1993; Cavender‐Bares & Pahlich, 2009; Chung et al., 2002; Craft & Ashley, 2006; Gomory et al., 2001; Kim et al., 1993; Mattila et al., 1994).

Related researchers in China have also conducted genetic diversity studies on some native oak varieties. Li, Chen, and Li (1997), Li, Chen, and Li (1998) studied the genetic structure of *Q. aquifolioides* and *Q. tungmaiensis* populations. Yun, Wang, et al. (1998), Yun, Zhong, et al. (1998) and Li, Gu, and Zhou (2003) analyzed the populations of *Q. liaotungensis* and *Q. mongolica*. Zhou, Guo, Yang, and Zhang (2003), Zhou, Lu, Zhang, and Zhang (2008) studied the population of *Q. variabilis*. The results also indicate that the genetic variation of native oak populations mainly exists within the population, the level of genetic variation among the populations is lower, and the gene exchange between the populations is frequent; compared with foreign oaks, the genetic diversity of some native oaks in China is lower (Greef, Triest, Cuyper, & Slycken, 1998; Hamrick, Godt, & Sherman‐Broles, 1992; Kremer & Petit, 1993; Li et al., 2003; Wang, Hu, & Zhou, 2005). For *Q. fabri*, the latest related research is to develop 12 polymorphic and 2 monomorphic microsatellite loci, and the genetic diversity of *Q. fabri* has not been studied in depth (Xiao, Chen, Bao, Wang, & Li, 2016).

In recent years, with the application and promotion of next‐generation sequencing (NGS), the simplified genome sequencing technology genotyping‐by‐sequencing (GBS) has gradually come to be used to analyze the genetic evolution of multiple species (Wu & Blair, 2017). GBS technology can quickly identify a considerable number of high‐density SNP markers, and these SNP markers can be used for genetic map construction, genome‐wide association analysis, phylogenetic analysis, and diversity analysis (Lee et al., 2019; Siadjeu, Mayland‐Quellhorst, & Albach, 2018). In addition, GBS technology can also be used for species with and without reference genomes. At present, GBS technology is becoming increasingly prevalent in the analysis of genetic diversity of plants and animals such as oil palm (*Elaeis guineensis*) (Xia et al., 2019), pepper (*Capsicum* spp.) (Pereira‐Dias, Vilanova, Fita, Prohens, & Rodríguez‐Burruezoet, 2019), *Dendrobium* (Ryu et al., 2019), and *Misgurnus anguillicaudatus* (Yi, Wang, & Zhou, 2019).

Considering that the genetics of *Q. fabri* are poorly understood despite its important uses, a diversity study of a collection of 158 *Q. fabri* individuals from 13 natural populations in China was presented using GBS. Our goals are to assess the population structure, genetic diversity, and relationships of *Q. fabri*.

## 2 MATERIALS AND METHODS

### 2.1 Plant material

**Figure 1:**
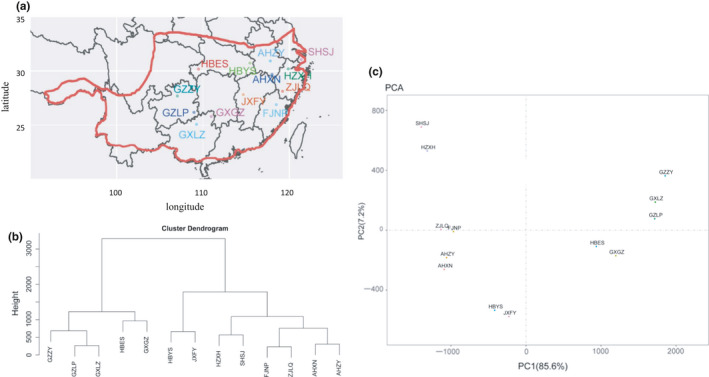
Sampling map of *Quercus fabri* in China. (a) Map showing the sampling locations. The natural distribution range of *Quercus fabri* has been marked with a red line. (b) Cluster analysis of the sampling locations. (c) PCA clustering of the sampling locations

In total, 158 individuals of *Q. fabri* were used in this study. All of these individuals were collected from 13 natural distribution areas of *Q. fabri* in China (Figure 1, Tables 1 and 2).

**Table 1:** The latitude and longitude of 13 sampling locations of *Quercus fabri*

**population**	**Latitude**	**Longitude**
**HBYS**	30^°^44′11″	115^°^40′46″
**HBES**	30^°^39′00″	110^°^4′12″
**JXFY**	27^°^82′11″	114^°^67′11″
**AHXN**	29^°^41′36″	118^°^0′54″
**AHZY**	30^°^56′6″	117^°^48′28″
**HZXH**	30^°^12′51″	120^°^7′50″
**FJNP**	27^°^22′12″	118^°^51′36″
**GZZY**	27^°^42′40″	107^°^02′55″
**GZLP**	26^°^11′24″	108^°^57′00″
**GXGZ**	25^°^48′00″	111^°^18′00″
**GXLZ**	25^°^4′12″	109^°^15′00″
**SHSJ**	31^°^3′10″	121^°^23′16″
**ZJLQ**	28^°^8′11″	119^°^13′11″

**Table 2:** Matrix of geographic distance (km) between pairwise sampling locations of *Quercus fabri*

**Origins**	**HBYS**	**HBES**	**JXFY**	**AHXN**	**AHZY**	**HZXH**	**FJNP**	**GZZY**	**GZLP**	**GXGZ**	**GXLZ**	**SHSJ**	**ZJLQ**
HBYS	0												
HBES	536.3	0											
JXFY	268.8	550.5	0										
AHXN	252.9	771.0	317.6	0									
AHZY	204.4	739.6	385.9	139.9	0								
HZXH	839.2	965.3	527.4	211.9	236.3	0							
FJNP	485.5	928.9	384.1	270.9	409.5	339.8	0						
GZZY	902.4	439.3	795.4	1,091.7	1,102.5	1,302.0	1,164.8	0					
GZLP	829.4	508.0	656.0	971.5	1,012.8	1,182.7	992.2	253.2	0				
GXGZ	696.4	552.7	473.8	789.6	854.9	995.6	771.5	472.7	239.0	0			
GXLZ	892.0	625.8	688.6	1,006.0	1,063.0	1,214.0	991.8	366.5	128.1	221.3	0		
SHSJ	545.7	1,080.7	674.7	357.1	341.4	152.2	477.4	1,437.0	1,327.8	1,145.5	1,362.9	0	
ZJLQ	447.8	928.2	401.5	208.3	339.7	247.9	91.9	1,195.4	1,037.5	825.6	1,046.9	386.7	0

### 2.2 DNA extraction, library preparation, and sequencing

Young leaves from 158 individuals of *Q. fabri* were collected, and the genomic DNA was extracted using a plant genomic DNA extraction kit (TIANGEN BIOTECH, Beijing, China). The purity of the extracted DNA was detected using a NanoDrop^®^ spectrophotometer (ND‐1000, Thermo Fisher Scientific, Wilmington, NC, USA), and DNA electrophoresis was simultaneously performed in a 1% agarose gel to ensure DNA integrity. A Qubit^™^ 2.0 Fluorometer (Invitrogen, Carlsbad, CA, USA) was used to accurately measure the DNA concentration. High‐quality DNA was used for GBS library construction and sequencing.

A total of 1.5 µg of DNA from each sample were used for library construction. The enzyme digestion evaluation was based on the *Quercus robur* reference genome, and the capture of 10w tags was used as the evaluation standard. Three different enzyme digestion protocols were selected for the evaluation, namely EcoRI and MseI, HaeII and MseI, and MspI and MseI. The results of enzyme digestion showed that the number of tags captured by HaeII and MseI was the largest and the coverage of the reference genome was higher (Table S1). Thus, the restriction enzymes MseI and HaeII (New England Biolabs, NEB) were used to digest the genomic DNA. The Illumina sequencing adapters and sample‐specific barcodes were ligated to both ends of the digested DNA fragment using T4 DNA ligase (New England Biolabs, NEB), and each sample was subjected to DNA fragment amplification and pooled. The 350–400 bp DNA fragments were recycled by agarose gel electrophoresis and purified. The purified DNA fragments were subjected to paired‐end 150 bp sequencing on the Illumina HiSeq sequencing platform.

### 2.3 GBS data analysis and SNP calling

The sequencing data were divided into corresponding samples according to their barcode, and all the raw reads were subjected to strict quality control screening to remove adapters and low‐quality short reads, including those where the “N” residues accounted for more than 10% of the read, or the percentage of low‐quality (*Q* ≤ 5) was greater than 50% in the read in single‐end sequencing. The selected high‐quality clean reads were aligned against the *Quercus robur* reference genome (https://www.ncbi.nlm.nih.gov/bioproject/PRJEB19898) using the Burrows‐Wheeler Aligner (BWA version 0.7.8‐r455) set to the default settings. The data mapping rate ranged from 84.78% to 96.19%, and the coverage 1X ranged from 9% to 15.62%. The alignment result was good, and the reference sequence was available (Table S2). SAMtools (version 1.8) was used to calculate the number of reads that passed quality control criteria and were successfully aligned to the reference. The percentage of sequence tags that overlapped with gene regions was assessed by applying BEDtools (version 2.25.0). Then, the aligned sequence tags were converted into BAM format for storage in a TOPM (TagsOnPhysicallMap) file. SNP calling was also performed with SAMtools. The high‐quality SNPs were obtained by filtering out low‐quality SNPs on the basis of the minimum minor allele frequency (mnMAF < 0.01) and the missing data per site (MDpS > 10%). The transitions/transversions (ts/tv) ratio was calculated using BCFtools (version 1.8).

### 2.4 Principal component analysis

The preliminary analysis of the population structure was performed by principal component analysis (PCA) using GCTA (version 1.26.0) based on the high‐quality SNPs. PC1 and PC2 as well as PC1 and PC3 were plotted against each other, with individuals assigned to different sample sites.

### 2.5 Population structure analysis

Use ADMIXTURE 1.23 to assign each sample to the most likely cluster using different numbers of clusters (*K* values) (Alexander, Novembre, & Lange, 2009). A population structure analysis of 1–10 clusters was set up (*K* = 1, 2, 3,…, 10), and the cross‐validation error (CV error) calculated by ADMIXTURE 1.23 with the sum of the values of 10 permutations was used to evaluate the most likely number of clusters.

### 2.6 Phylogenetic tree construction

A phylogenetic tree was constructed using the neighbor‐joining method in the MEGA program (version 7.0) based on a pairwise genetic distance matrix of 158 individuals, which was obtained using TreeBest (version 1.9.2) with 1,000 bootstrap replicates (Kumar, Stecher, & Tamura, 2016).

### 2.7 Genetic diversity index analysis

The genetic diversity includes expected heterozygosity (*He*) and observed heterozygosity (*Ho*) analyses, and analysis of molecular variance (AMOVA). Expected heterozygosity could be calculated as described by Harris and DeGiorgio under Hardy–Weinberg equilibrium (Harris & DeGiorgio, 2017). Observed heterozygosity was calculated using GenAlEx (version 6.503) (Peakall & Smouse, 2012). AMOVA is used to calculate the proportion of the variation between populations or within populations within the total variation. The number of groups determined with ADMIXTURE 1.23 was used for AMOVA in GenAlEx (version 6.503) (Peakall & Smouse, 2012).

## 3 RESULTS

### 3.1 GBS data analysis summary

A collection of 158 *Q. fabri* individuals were successfully sequenced using the Illumina HiSeq sequencing platform, and an average of 5.07 million clean reads per *Q. fabri* individuals was generated with an average depth per individual of 9.53 (Table S2). The percentage of GC content was approximately 40%, which was within the normal range. The sequencing quality was high (Q20 ≥ 90%, Q30 ≥ 85%), so subsequent analyses could be performed (Table S3). The sequencing result for each individual was aligned with the reference genome, and the similarity between them reached the requirement for resequencing analysis. The average degree of sequence coverage was 11.89% for at least single‐base coverage, and the average degree of coverage with at least 4‐base coverage was 7.78% (Table S2). A total of 10,402,236 SNPs were obtained according to detection with SAMtools software, and 459,564 high‐quality SNPs were obtained after filtration for subsequent analysis. Among these high‐quality SNPs, the number of SNPs belonging to base transitions was 2.808 times the number of SNPs belonging to base transversions, indicating that most of the SNPs were of the base transition type (Table S4).

### 3.2 Genetic structure and phylogenetic analysis

**Figure 2:**
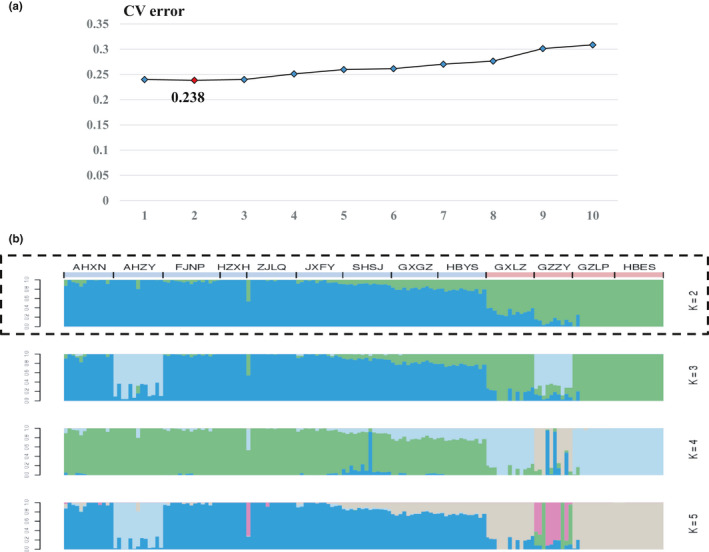
Genetic structure analysis of 158 *Quercus fabri* individuals. (a) The curve of cross‐validation error (CV error) values under different K values. Red diamond represents the most probable true K value. (b) Genetic structure of 158 *Quercus fabri* individuals under different *K* values. Each vertical bar represents one individual, and the colors represent ancestry components. Dashed line represents the most likely number of clusters. The area indicated by the pink strip is group 1, and the area indicated by the blue strip is group 2

We used ADMIXTURE to study genetic relationships and population structure among the 158 *Q. fabri* individuals that originating from 13 populations of *Q. fabri* in China. The most likely number of clusters was evaluated by the cross‐validation error as *K* = 2 (CV error = 0.238), and the error was lower than *K* = 1 and *K* = 3 (CV error = 0.240 and 0.240, respectively; Figure 2a). We used the criterion to group each individual: When at least 60% of its inferred pedigree came from a certain group, we assigned it to the group. The grouping results showed that the group 1 contained the populations in West China (HBES), Southwest China (GZZY and GZLP), and South China (GXLZ); the group 2 mainly contained those from Central China (HBYS and JXFY) and East China (AHXN, AHZY, FJNP, HZXH, ZJLQ, and SHSJ); in addition, it also includes the GXGZ population from South China (Figure 2b). From Figure 2b, we could find that the genetic structures of GXGZ and HBYS were very similar, and it could be seen that within their genetic structures, the genetic ratio of ancestry in East China was significantly higher than that of ancestry in Southwestern China (Figure 2b). These results suggest that differences in geographic origin may be one of the important causes of genetic differences in *Q. fabri*.

**Figure 3:**
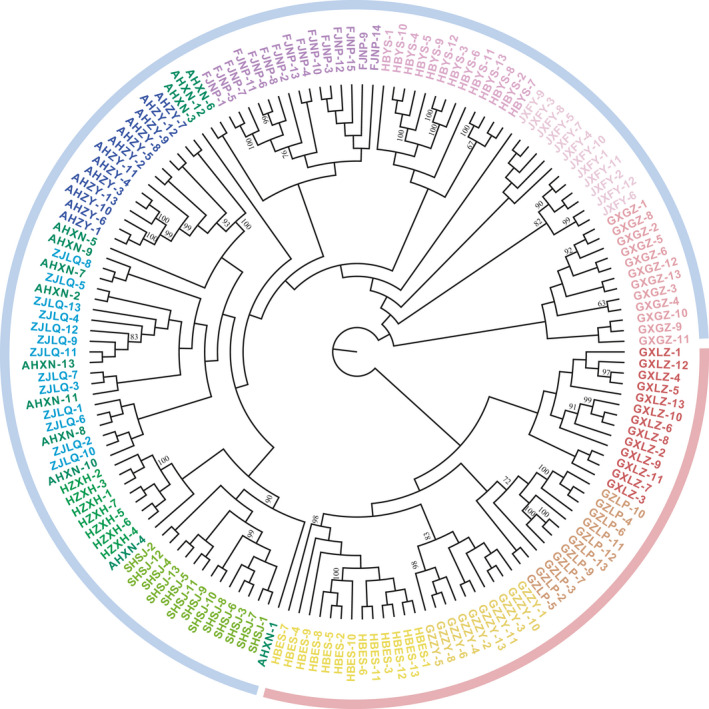
The neighbor‐joining tree of 158 *Quercus fabri* individuals. Branches are colored to distinguish different provenances. The area indicated by the pink strip is group 1, and the area indicated by the blue strip is group 2. Node values correspond to bootstrap values

**Figure 4:**
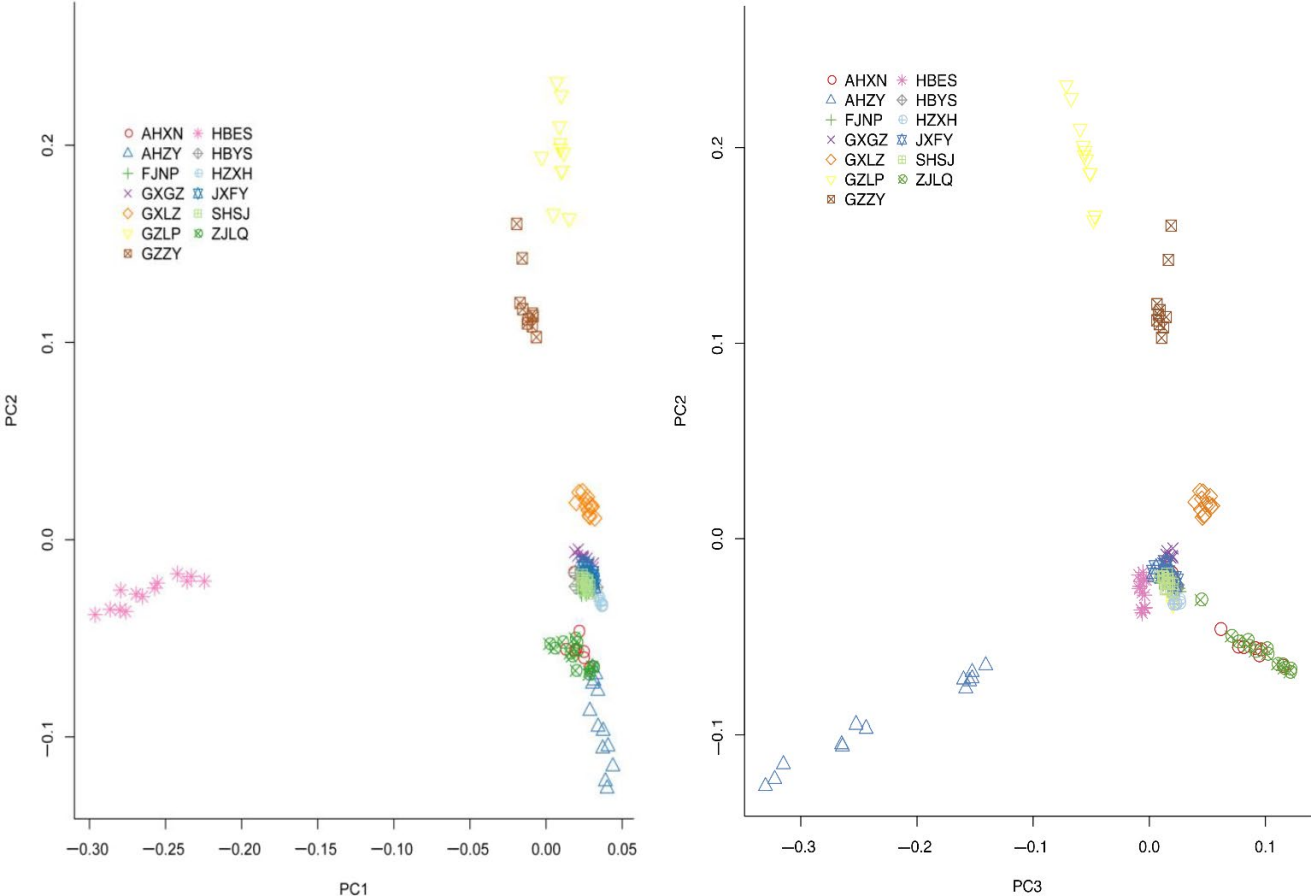
Principal component analysis (PCA) of 158 *Quercus fabri* individuals

Consistent with the results of the population structure analysis, the phylogenetic analysis conducted by the MEGA program to establish neighbor‐joining tree also divided 158 *Q. fabri* individuals into two main groups (Figure 3). In addition, all individuals from the same sampling area were clustered together except the AHXN population, in which the individuals were scattered among the FJNP, AHZY, ZJLQ, HZXH, and SHSJ populations. The JXFY and HBYS populations were each relatively independent but were located slightly closer to the FJNP population (Figure 3). From the result of phylogenetic analysis, there may be different evolution paths for *Q. fabri* in West China, Southwest China, South China, and East China. There are close genetic relationships among several populations from plateau and mountainous areas of West China, Southwest China, and South China, and there are also close genetic relationships among several populations distributed in the middle and lower reaches of the Yangtze River. In addition, consistent with the results of population structure and phylogenetic analysis, the distribution of all 158 *Q. fabri* individuals produced by the principal component analysis also divided all individuals into two groups (Figure 4).

### 3.3 Population genetic diversity analysis

A genetic divergence was observed in the two groups and expected heterozygosity (*He*) among individuals in each group (Table 3). The higher value of expected heterozygosity was found in group 2 with a value of 0.170, and the lower value was found in group 1 with a value of 0.163. However, the higher value of observed heterozygosity was found in group 1 with a value of 0.093, and the lower value was found in group 2 with a value of 0.090 (Table 3). The AMOVA revealed that 12.36% of the total variation occurred between the two groups while the rest (87.64%) occurred within groups, indicating significant genetic variation within groups (*p* < .001).

**Table 3:** Summary statistics of different genetic parameters including observed heterozygosity (*Ho*), expected heterozygosity (*He*), and number of individuals in each group of *Quercus fabri*

**Group**	***Ho***	***He***	**No. of Individuals**
1	0.093	0.163	47
2	0.090	0.170	111

## 4 DISCUSSION

*Quercus fabri* Hance is specifically distributed in various subtropical provinces in China and is often grown in the woods of hills and mountainous areas at an altitude of 50–1900 meters. Although *Q. fabri* is extremely important in wood production and forest by‐product production, the *Q. fabri* populations in China are declining. Therefore, it is necessary to carry out research work on genetic diversity analysis, germplasm collection, evaluation, and conservation. In this study, we obtained 459,564 high‐quality SNPs for subsequent analysis using GBS technology and found that the number of transition SNPs was significantly more than transversions in *Q. fabri*, suggesting that during natural selection, individuals with transition mutations were more adaptive than individuals with transversion mutations (Luo, Iaffaldano, Zhuang, Fresnedo‐Ramirez, & Cornish, 2017). This result may also be due to the large number of base transitions that have caused only synonymous mutations in the sequence encoding the protein (Guo et al., 2017).

### 4.1 Population genetic structure of *Quercus fabri* Hance

The ADMIXTURE analyses allowed the assignment of populations to groups based on their genetic similarities and geographic isolation and provided information about the proportions of various ancestors in the genetic structure. Our assignment test results based on ADMIXTURE separate *Q. fabri* populations into two groups, and the geographical environment and climatic characteristics of the two groups are very different.

The terrain of group 1 distribution belongs to the transition area from plateau to hills. There are many mountains in the territory, and the mountains are overlapping, with an average altitude of about 1,100 m. The Wushan Mountains in the north of the distribution area, and the Wuling Mountains, Xuefeng Mountains, and Yuechengling Mountains in the middle and southeast, respectively, isolate the group 2 distribution area. The climate in group 1 distribution is warm, with small temperature changes, warm in winter and cool in summer; abundant precipitation and high humidity, belonging to a subtropical humid monsoon climate. The suitable climate makes the vegetation grow lush, and the forest coverage in the group 1 distribution area is extremely high, which may also be one of the main reasons for separating the two ancestors and preventing gene exchange. The group 2 distribution is mostly in the middle and lower reaches of the Yangtze River. The terrain is low and flat, and the altitude is mostly below 50 m. The temperature is also significantly higher than that of group 1 distribution areas. The differences in the genetic structure of *Q. fabri* in different regions may also be the changes in genetic information that they have to occur in order to adapt to different environments.

The differences in environment also lead to differences in population and consumption behavior. For example, the population of the eastern plains of China is significantly larger than that of the southwestern mountains, and because of the large number of metropolises in the eastern region, the economy is significantly more developed than the southwestern region, and the frequency of people's activities is also significantly higher, which may lead to the genetic information exchange between *Q. fabri* resources is more frequent, and the genetic structure among populations in group 2 is very similar. The genetic structure of several populations in the southwestern mountainous area of group 1, such as GXLZ, GZZY, and GZLP, is quite different, which may also be caused by mountain barriers and people's lower activity frequency. During this period, the main purpose of *Q. fabri* is to burn charcoal. In order to withstand the cold, people in the southwestern mountainous areas have cut down *Q. fabri* trees to burn, causing a sharp decline in the *Q. fabri* resources of this area. This result also suggests that we urgently need to strengthen the protection of *Q. fabri* resources.

The genetic structure analysis also showed that although the JXFY and HBYS populations are located in Central China, most of their genetic information came from East China (Figure 2). In addition, GXGZ population from South China was clustered into group 2 and most of its genetic information also came from East China. It revealed that the ancestors from East China have been moving westward, causing the majority of the genetic background of the GXGZ population to be the same as that of populations from East China (Figure 2). However, we found that a fraction of the genetic background of the HBYS and GXGZ populations was similar to that of the West China populations, indicating that the genetic structure of the two populations came from the fusion of two ancestors of the East China *Q. fabri* and the West China *Q. fabri* (Figure 2). In comparison, the proportion of the ancestral contribution from East China was higher in the HBYS and GXGZ populations, which suggests that the ancestral genetic information from coastal areas flows inward to inland areas significantly faster than the flow in the opposite direction (Figure 2). The reason for this difference may be that there is a special geographic structure in many high mountain areas in Southwest China, which has restricted gene flow from West China, Southwest China, and South China to East China.

Both phylogenetic analysis and principal component analysis showed consistent results with genetic structure analysis. The neighbor‐joining tree also revealed that the populations of *Q. fabri* could be clustered into two groups on the basis of the degree of their genetic relationships (Figure 3). For example, the HBES, GZZY, GZLP, and GXLZ populations were closely clustered into one group, suggesting a high degree of genetic relationship between these populations, which could be due to their locations in nearby geographic regions and possible high gene flow. Similarly, the SHSJ, HZXH, ZJLQ, AHXN, AHZY, FJNP, JXFY, HBYS, and GXGZ populations were closely clustered into one group, indicating that the genetic relationships between them are close, which is also related to their geographical locations (Figure 3).

In view of the large difference in genetic structure between the two groups, it is necessary for *Q. fabri* breeding programs to incorporate the other genetic basis by utilizing germplasm from the other group particularly from the population with the farthest geographical distance between them as breeding parents. Due to the potential interspecific hybridization between oak trees, it is necessary to introduce other oaks germplasm by artificial hybridization in the future in order to broaden the genetic basis of *Q. fabri* germplasm.

### 4.2 Genetic diversity of *Quercus fabri* Hance

Observed heterozygosity (*Ho*) is the probability that the alleles of two randomly selected samples are not the same and are the ratio of the number of heterozygous individuals observed to the total number of individuals in the sample. Expected heterozygosity (*He*) describes the expected proportion of heterozygous genotypes, which can be calculated according to the method provided by Harris and DeGiorgio (2017). Formally, *He* is the probability that a pair of randomly selected alleles from a population is different, which indicates the evolutionary pressure of the allele of an individual and the probability of a certain locus to mutate over a period of time (Shete, Tiwari, & Elston, 2000). In this study, we found that although both groups had similar *He*, group 2 was slightly higher than group 1, meaning that group 2 was more diverse than group 1 since *He* depends on both the number of alleles and the abundance of the alleles in a group.

It is generally believed that populations with a heterozygosity higher than 0.5 are not subject to high‐intensity manual selection or natural selection and have high genetic diversity. This study detected that the observed heterozygosity and expected heterozygosity of these *Q. fabri* populations are both less than 0.5. The reason may be mainly because *Q. fabri* is a self‐pollinating plant, and its male flowers have a sufficient amount of pollen and can often produce progeny through selfing, so the heterozygosity of *Q. fabri* is relatively low.

The results of AMOVA indicated high level of genetic diversity within groups and meant that the individual differences within groups are greater than the differences between the two groups. The potential interspecific hybridization between *Q. fabri* and other oaks in the same location was considered the main reason for the variation within groups. Through the field survey accompanying the sampling process, we found that there were many other oak resources located around these populations, such as *Quercus acutissima* Carruth., *Q. variabilis* Bl., *Q. aliena* Bl., and *Q. serrata* Thunb. Interspecific hybridization is likely to occur between *Quercus* species, resulting in genetic communication.

## 5 CONCLUSIONS

The application of GBS analysis for the assessment of population genetic diversity and genetic structure has become increasingly common and is usually necessary before decisions about the preservation and utilization of species resources are made. In this study, we analyzed the genetic diversity and genetic structure of 13 *Q. fabri* populations and found that these populations can be clustered into two groups and the genetic structure was explained mainly by their geographic location. The genetic relationship of adjacent populations was more similar, and infiltration of genetic background from coastal areas to inland areas was observed, where the hindrance of genetic infiltration by mountainous areas was obvious. In addition, the individual differences within groups are greater than the differences between the two groups, which benefits genetic improvement and breeding of *Q. fabri* cultivars.

## 6 DATA ARCHIVING STATEMENT

The datasets supporting the conclusions of this article are available in the NCBI Sequence Read Archive (SRA) database under accession number PRJNA564867. http://www.ncbi.nlm.nih.gov/bioproject/564867

## CONFLICT OF INTEREST

The authors declare no conflict of interest.

## AUTHOR CONTRIBUTION

**Shifa Xiong:** Writing‐original draft (lead). **Yunxiao Zhao:** Data curation (equal); Formal analysis (equal). **Yicun Chen:** Formal analysis (equal); Methodology (equal). **Ming Gao:** Data curation (equal); Methodology (equal). **Liwen Wu:** Funding acquisition (equal); Methodology (equal); Project administration (equal); Supervision (equal); Validation (equal); Writing‐review & editing (equal). **Yangdong Wang:** Supervision (lead); Validation (equal); Writing‐review & editing (equal).

## Supporting information

supinfo

## Data Availability

The datasets supporting the conclusions of this article are available in the NCBI Sequence Read Archive (SRA) database under accession number PRJNA564867. http://www.ncbi.nlm.nih.gov/bioproject/564867
