# Supplementary material for: Genetic diversity and population structure of Quercus fabri Hance in China revealed by genotyping‐by‐sequencing
Source: Ecol Evol. 2020 Jul 24;10(16):8949–58. doi: 10.1002/ece3.6598 (PMC7452768; doi:10.1002/ece3.6598)
Supplement: Supplementary file 1 — supinfo [file ECE3-10-8949-s001.docx]

**Supplementary data**

**Table S1.** Statistics of evaluation data of electronic enzyme digestion

| Genomic information | Scaffold length  790292631 bp | Program I | Program II | Program III |
| --- | --- | --- | --- | --- |
| Enzyme digestion protocol | Endonuclease | EcoRI+MseI | HaeII+MseI | MspI+MseI |
|  | Scope of fragments（bp） | 265-290 | 265-290 | 265-290 |
|  | Number of tag | 192192 | 201654 | 177532 |
|  | Genomic coverage | 3.3% | 3.47% | 3.05% |

**Table S2.** Statistics of sequencing depth and coverage of 158 *Quercus fabri* individuals.

| **Sample** | **Clean_reads** | **mapped_reads** | **mapping_rate** | **Average_depth** | **Coverage_1X** | **Coverage_4X** |
| --- | --- | --- | --- | --- | --- | --- |
| AHXN-1 | 5726092 | 5274558 | 92.11% | 9.48 | 12.88% | 8.54% |
| AHXN-2 | 5767280 | 5523168 | 95.77% | 9.22 | 14.01% | 9.22% |
| AHXN-3 | 4846528 | 4546592 | 93.81% | 8.98 | 12.04% | 7.79% |
| AHXN-4 | 5333316 | 4878498 | 91.47% | 10.12 | 11.34% | 7.45% |
| AHXN-5 | 3532024 | 3339310 | 94.54% | 9.17 | 9.00% | 5.53% |
| AHXN-6 | 5883480 | 5426892 | 92.24% | 10.45 | 12.05% | 8.07% |
| AHXN-7 | 5320400 | 5093762 | 95.74% | 10.09 | 11.95% | 7.98% |
| AHXN-8 | 5623000 | 5383194 | 95.74% | 9.77 | 12.78% | 8.51% |
| AHXN-9 | 6075044 | 5811250 | 95.66% | 10.56 | 12.44% | 8.38% |
| AHXN-10 | 6220448 | 5930260 | 95.33% | 10.65 | 12.82% | 8.72% |
| AHXN-11 | 6536372 | 6171300 | 94.41% | 9.66 | 14.52% | 9.95% |
| AHXN-12 | 6187628 | 5603102 | 90.55% | 9.24 | 14.04% | 9.49% |
| AHXN-13 | 5581300 | 5283096 | 94.66% | 8.75 | 13.80% | 9.20% |
| AHZY-1 | 5573060 | 5344956 | 95.91% | 8.53 | 14.35% | 9.57% |
| AHZY-2 | 5733212 | 5492106 | 95.79% | 8.94 | 14.18% | 9.50% |
| AHZY-3 | 5790824 | 5553186 | 95.90% | 9.11 | 14.03% | 9.35% |
| AHZY-4 | 5356064 | 5139524 | 95.96% | 10.02 | 12.12% | 8.00% |
| AHZY-5 | 6077736 | 5843386 | 96.14% | 9.97 | 13.36% | 9.06% |
| AHZY-6 | 6357956 | 6094980 | 95.86% | 10.66 | 13.26% | 9.04% |
| AHZY-7 | 5119528 | 4911424 | 95.94% | 9.87 | 11.87% | 7.63% |
| AHZY-8 | 6087808 | 5843060 | 95.98% | 10.51 | 12.89% | 8.69% |
| AHZY-9 | 6417644 | 6161700 | 96.01% | 10.5 | 13.64% | 9.19% |
| AHZY-10 | 3871500 | 3689056 | 95.29% | 8.54 | 10.51% | 6.37% |
| AHZY-11 | 4527188 | 4328262 | 95.61% | 8.85 | 11.80% | 7.46% |
| AHZY-12 | 5066472 | 4828334 | 95.30% | 8.71 | 13.01% | 8.36% |
| AHZY-13 | 4640004 | 4436510 | 95.61% | 9.29 | 11.33% | 7.19% |
| FJNP-1 | 5070304 | 4828510 | 95.23% | 10.13 | 11.28% | 7.29% |
| FJNP-2 | 5250088 | 5021104 | 95.64% | 10.32 | 11.42% | 7.50% |
| FJNP-3 | 4641840 | 4436376 | 95.57% | 9.43 | 11.31% | 7.23% |
| FJNP-4 | 4512112 | 4303256 | 95.37% | 9.93 | 10.63% | 6.68% |
| FJNP-5 | 4850988 | 4639248 | 95.64% | 9.81 | 11.22% | 7.24% |
| FJNP-6 | 5647848 | 5399960 | 95.61% | 10.05 | 12.67% | 8.41% |
| FJNP-7 | 4271536 | 4093602 | 95.83% | 9.15 | 10.77% | 6.77% |
| FJNP-8 | 4445504 | 4250656 | 95.62% | 8.51 | 11.94% | 7.55% |
| FJNP-9 | 5001988 | 4788178 | 95.73% | 9 | 12.52% | 8.14% |
| FJNP-10 | 4498916 | 4317656 | 95.97% | 9.31 | 11.06% | 7.08% |
| FJNP-11 | 5104316 | 4890746 | 95.82% | 9.41 | 12.20% | 8.04% |
| FJNP-12 | 4038288 | 3868418 | 95.79% | 8.75 | 10.66% | 6.72% |
| FJNP-13 | 5116000 | 4905992 | 95.90% | 10.06 | 11.64% | 7.63% |
| FJNP-14 | 3499368 | 3366080 | 96.19% | 8.63 | 9.59% | 5.80% |
| FJNP-15 | 4970056 | 4763616 | 95.85% | 10 | 11.40% | 7.48% |
| GXGZ-1 | 5355592 | 5128568 | 95.76% | 9.22 | 13.08% | 8.61% |
| GXGZ-2 | 5925556 | 5686354 | 95.96% | 10 | 13.15% | 8.76% |
| GXGZ-3 | 6583912 | 6318630 | 95.97% | 10.44 | 13.96% | 9.46% |
| GXGZ-4 | 5520064 | 5288944 | 95.81% | 9.64 | 12.97% | 8.57% |
| GXGZ-5 | 6097860 | 5848700 | 95.91% | 10.05 | 13.56% | 9.17% |
| GXGZ-6 | 5919264 | 5680412 | 95.96% | 9.63 | 13.65% | 9.20% |
| GXGZ-8 | 6086596 | 5849804 | 96.11% | 9.97 | 13.68% | 9.20% |
| GXGZ-9 | 6128044 | 5871804 | 95.82% | 9.88 | 13.87% | 9.38% |
| GXGZ-10 | 5523752 | 5309050 | 96.11% | 9.32 | 13.32% | 8.81% |
| GXGZ-11 | 7287284 | 7002306 | 96.09% | 11.96 | 13.36% | 9.14% |
| GXGZ-12 | 6466924 | 6213358 | 96.08% | 10.65 | 13.26% | 9.05% |
| GXGZ-13 | 5765632 | 5532226 | 95.95% | 9.6 | 13.58% | 9.00% |
| GXLZ-1 | 4965160 | 4724918 | 95.16% | 9.29 | 11.89% | 7.90% |
| GXLZ-2 | 5239824 | 4993548 | 95.30% | 9.77 | 11.91% | 7.92% |
| GXLZ-3 | 4768652 | 4556138 | 95.54% | 9.18 | 11.66% | 7.68% |
| GXLZ-4 | 4284624 | 4097438 | 95.63% | 8.76 | 11.03% | 7.07% |
| GXLZ-5 | 5137956 | 4914906 | 95.66% | 9.66 | 11.71% | 7.81% |
| GXLZ-6 | 4316748 | 4116816 | 95.37% | 8.72 | 11.11% | 7.12% |
| GXLZ-7 | 4639524 | 4425658 | 95.39% | 9.33 | 11.24% | 7.33% |
| GXLZ-8 | 4852400 | 4622476 | 95.26% | 9.96 | 10.95% | 7.22% |
| GXLZ-9 | 4876924 | 4657968 | 95.51% | 9.3 | 11.62% | 7.73% |
| GXLZ-10 | 4367116 | 4162806 | 95.32% | 9.35 | 10.56% | 6.85% |
| GXLZ-11 | 4889552 | 4644078 | 94.98% | 10.02 | 11.06% | 7.27% |
| GXLZ-12 | 4607796 | 4389496 | 95.26% | 9.12 | 11.24% | 7.32% |
| GXLZ-13 | 4959736 | 4739686 | 95.56% | 9.43 | 11.73% | 7.78% |
| GZLP-2 | 5742904 | 5504848 | 95.85% | 10.34 | 12.41% | 8.34% |
| GZLP-3 | 4934508 | 4701522 | 95.28% | 9.45 | 11.62% | 7.63% |
| GZLP-4 | 4564320 | 4341484 | 95.12% | 9.11 | 11.30% | 7.36% |
| GZLP-5 | 4308644 | 4111904 | 95.43% | 9 | 10.91% | 6.96% |
| GZLP-6 | 4855184 | 4629956 | 95.36% | 9.35 | 11.64% | 7.64% |
| GZLP-7 | 4341896 | 4134026 | 95.21% | 8.4 | 11.67% | 7.50% |
| GZLP-9 | 4271960 | 4059154 | 95.02% | 9.03 | 10.72% | 6.88% |
| GZLP-10 | 4733508 | 4514698 | 95.38% | 9.25 | 11.41% | 7.53% |
| GZLP-11 | 4246196 | 4045054 | 95.26% | 8.87 | 10.84% | 6.90% |
| GZLP-12 | 4145572 | 3943304 | 95.12% | 8.92 | 10.57% | 6.73% |
| GZLP-13 | 4829880 | 4565060 | 94.52% | 9.09 | 11.84% | 7.73% |
| GZZY-1 | 5036612 | 4836592 | 96.03% | 10.21 | 11.35% | 7.40% |
| GZZY-2 | 4634948 | 4452600 | 96.07% | 9.62 | 11.14% | 7.16% |
| GZZY-3 | 5732552 | 5510002 | 96.12% | 11.15 | 11.63% | 7.64% |
| GZZY-4 | 5135424 | 4919428 | 95.79% | 9.33 | 12.46% | 8.17% |
| GZZY-5 | 4075376 | 3902322 | 95.75% | 8.39 | 11.25% | 7.01% |
| GZZY-6 | 4163364 | 3989956 | 95.83% | 8.86 | 10.92% | 6.85% |
| GZZY-8 | 4029540 | 3860930 | 95.82% | 8.64 | 10.76% | 6.73% |
| GZZY-10 | 4133428 | 3960120 | 95.81% | 8.9 | 10.72% | 6.73% |
| GZZY-11 | 4717156 | 4530034 | 96.03% | 9.53 | 11.41% | 7.39% |
| GZZY-13 | 4692720 | 4491326 | 95.71% | 9.92 | 10.99% | 7.09% |
| HBES-1 | 4299628 | 4115070 | 95.71% | 9.19 | 10.78% | 6.96% |
| HBES-2 | 5177212 | 4960666 | 95.82% | 10.15 | 11.23% | 7.47% |
| HBES-3 | 4845260 | 4597438 | 94.89% | 10.24 | 10.72% | 7.11% |
| HBES-4 | 5350584 | 5125446 | 95.79% | 10.31 | 11.50% | 7.72% |
| HBES-5 | 5256160 | 5007996 | 95.28% | 9.91 | 11.63% | 7.76% |
| HBES-6 | 4996384 | 4762052 | 95.31% | 9.98 | 11.13% | 7.29% |
| HBES-7 | 4168984 | 3934514 | 94.38% | 8.87 | 10.54% | 6.76% |
| HBES-8 | 4877300 | 4665336 | 95.65% | 9.88 | 10.94% | 7.23% |
| HBES-9 | 4568364 | 4371480 | 95.69% | 9.92 | 10.58% | 6.94% |
| HBES-10 | 4503772 | 4290852 | 95.27% | 9.99 | 10.19% | 6.54% |
| HBES-11 | 4909764 | 4708328 | 95.90% | 11.79 | 9.55% | 6.27% |
| HBES-12 | 5453656 | 5088546 | 93.31% | 11.36 | 10.49% | 7.15% |
| HBES-13 | 4998704 | 4787016 | 95.77% | 9.47 | 12.09% | 7.81% |
| HBYS-1 | 4706492 | 4497844 | 95.57% | 9.51 | 11.01% | 7.23% |
| HBYS-2 | 4924476 | 4710502 | 95.65% | 9.44 | 11.57% | 7.73% |
| HBYS-3 | 4551140 | 4363110 | 95.87% | 9.16 | 11.10% | 7.26% |
| HBYS-4 | 5017952 | 4804154 | 95.74% | 9.38 | 11.71% | 7.78% |
| HBYS-5 | 3863644 | 3692282 | 95.56% | 8.77 | 10.09% | 6.35% |
| HBYS-6 | 4515044 | 4303628 | 95.32% | 9.42 | 10.85% | 7.02% |
| HBYS-7 | 4769924 | 4563444 | 95.67% | 9.93 | 10.53% | 6.92% |
| HBYS-8 | 4818704 | 4608422 | 95.64% | 9.68 | 10.96% | 7.30% |
| HBYS-9 | 4384028 | 4191010 | 95.60% | 9.97 | 10.01% | 6.50% |
| HBYS-10 | 4499900 | 4294386 | 95.43% | 10.02 | 10.06% | 6.51% |
| HBYS-11 | 4255484 | 4076266 | 95.79% | 9.31 | 10.34% | 6.72% |
| HBYS-12 | 5014916 | 4802464 | 95.76% | 9.71 | 11.28% | 7.48% |
| HBYS-13 | 4190256 | 4004486 | 95.57% | 9.61 | 9.80% | 6.25% |
| HZXH-1 | 4895056 | 4671930 | 95.44% | 8.97 | 12.16% | 7.84% |
| HZXH-2 | 4546040 | 4337726 | 95.42% | 8.55 | 11.88% | 7.55% |
| HZXH-3 | 4458776 | 4259940 | 95.54% | 9.4 | 10.79% | 6.83% |
| HZXH-4 | 3877820 | 3719154 | 95.91% | 8.53 | 10.52% | 6.47% |
| HZXH-5 | 5257860 | 5025932 | 95.59% | 9.81 | 11.98% | 7.82% |
| HZXH-6 | 5206728 | 4985726 | 95.76% | 9.82 | 11.72% | 7.64% |
| HZXH-7 | 4823152 | 4616722 | 95.72% | 9.66 | 11.30% | 7.28% |
| JXFY-1 | 4773656 | 4585922 | 96.07% | 8.5 | 12.68% | 8.17% |
| JXFY-2 | 5576104 | 5345034 | 95.86% | 9.15 | 13.49% | 8.95% |
| JXFY-3 | 4115844 | 3950846 | 95.99% | 8.72 | 10.76% | 6.72% |
| JXFY-4 | 4146800 | 3974458 | 95.84% | 8 | 11.89% | 7.41% |
| JXFY-5 | 4958272 | 4763452 | 96.07% | 8.87 | 12.41% | 8.07% |
| JXFY-6 | 3784608 | 3635084 | 96.05% | 8.83 | 10.07% | 6.16% |
| JXFY-7 | 4186504 | 4018494 | 95.99% | 9.1 | 10.74% | 6.83% |
| JXFY-8 | 3933624 | 3774352 | 95.95% | 8.91 | 10.31% | 6.39% |
| JXFY-9 | 4938336 | 4747690 | 96.14% | 9.79 | 11.26% | 7.36% |
| JXFY-10 | 5336440 | 5127660 | 96.09% | 9.64 | 12.35% | 8.12% |
| JXFY-11 | 5864060 | 5630642 | 96.02% | 10.85 | 12.40% | 8.03% |
| JXFY-12 | 5699848 | 5467732 | 95.93% | 9.13 | 13.65% | 9.04% |
| SHSJ-1 | 5848544 | 5607274 | 95.87% | 8.74 | 14.92% | 10.02% |
| SHSJ-2 | 5333016 | 5112474 | 95.86% | 8.51 | 14.05% | 9.22% |
| SHSJ-3 | 6324500 | 6065792 | 95.91% | 9.2 | 15.18% | 10.31% |
| SHSJ-4 | 6334336 | 6071396 | 95.85% | 8.83 | 15.62% | 10.61% |
| SHSJ-5 | 6423844 | 6143898 | 95.64% | 10.24 | 14.02% | 9.50% |
| SHSJ-6 | 6459752 | 6194356 | 95.89% | 10.93 | 13.10% | 8.97% |
| SHSJ-7 | 6919844 | 6636238 | 95.90% | 9.81 | 15.40% | 10.66% |
| SHSJ-8 | 6073152 | 5824490 | 95.91% | 9.74 | 14.08% | 9.34% |
| SHSJ-9 | 6094220 | 5834820 | 95.74% | 10.35 | 13.14% | 8.84% |
| SHSJ-10 | 6363788 | 6117062 | 96.12% | 9.98 | 14.06% | 9.50% |
| SHSJ-11 | 6071096 | 5832716 | 96.07% | 9.39 | 14.16% | 9.52% |
| SHSJ-12 | 4511256 | 4327774 | 95.93% | 9.8 | 10.66% | 6.72% |
| SHSJ-13 | 5013488 | 4817928 | 96.10% | 9.49 | 11.87% | 7.70% |
| ZJLQ-1 | 4806640 | 4523748 | 94.11% | 9.49 | 11.30% | 7.27% |
| ZJLQ-2 | 5004708 | 4725898 | 94.43% | 9.65 | 11.52% | 7.46% |
| ZJLQ-3 | 3976112 | 3439196 | 86.50% | 8.79 | 9.64% | 5.92% |
| ZJLQ-4 | 5403572 | 5099520 | 94.37% | 9.85 | 12.17% | 8.08% |
| ZJLQ-5 | 5313000 | 4504552 | 84.78% | 9.9 | 10.96% | 7.07% |
| ZJLQ-6 | 5388976 | 4925110 | 91.39% | 10.22 | 11.56% | 7.46% |
| ZJLQ-7 | 5390704 | 5126836 | 95.11% | 9.92 | 12.19% | 8.02% |
| ZJLQ-8 | 5627236 | 5180634 | 92.06% | 9.67 | 12.23% | 8.05% |
| ZJLQ-9 | 4808220 | 4441320 | 92.37% | 9.37 | 11.23% | 7.24% |
| ZJLQ-10 | 4054876 | 3833920 | 94.55% | 8.77 | 10.60% | 6.62% |
| ZJLQ-11 | 4592756 | 4379370 | 95.35% | 8.61 | 12.01% | 7.66% |
| ZJLQ-12 | 5034772 | 4303028 | 85.47% | 9.02 | 11.28% | 7.23% |
| ZJLQ-13 | 4755692 | 4065610 | 85.49% | 8.94 | 11.05% | 6.89% |
| Mean | 5071447 | 4827123 | 95.17% | 9.53 | 11.89% | 7.78% |

**Table S3.** Quality statistics of sequencing data of 158 *Quercus fabri* individuals.

| Sample | Raw Base(bp) | Clean Base(bp) | Effective Rate(%) | Error Rate(%) | Q20(%) | Q30(%) | GC Content(%) |
| --- | --- | --- | --- | --- | --- | --- | --- |
| AHXN-1 | 412,305,984 | 412,278,624 | 99.99 | 0.03 | 95.53 | 88.07 | 39.32 |
| AHXN-2 | 415,286,208 | 415,244,160 | 99.99 | 0.03 | 95.92 | 89.22 | 37.97 |
| AHXN-3 | 348,970,176 | 348,950,016 | 99.99 | 0.03 | 96.46 | 90.67 | 38.65 |
| AHXN-4 | 384,020,928 | 383,998,752 | 99.99 | 0.03 | 96.2 | 89.88 | 39.08 |
| AHXN-5 | 254,338,560 | 254,305,728 | 99.99 | 0.04 | 94.22 | 86.59 | 36.53 |
| AHXN-6 | 423,610,848 | 423,610,560 | 100 | 0.03 | 96.77 | 91.32 | 39.09 |
| AHXN-7 | 383,092,416 | 383,068,800 | 99.99 | 0.03 | 96.38 | 90.31 | 38.8 |
| AHXN-8 | 404,856,288 | 404,856,000 | 100 | 0.03 | 95.19 | 87.37 | 39.08 |
| AHXN-9 | 437,403,744 | 437,403,168 | 100 | 0.03 | 95.69 | 88.56 | 39.57 |
| AHXN-10 | 447,872,544 | 447,872,256 | 100 | 0.03 | 95.94 | 89.22 | 38.95 |
| AHXN-11 | 470,647,296 | 470,618,784 | 99.99 | 0.03 | 95.12 | 87.23 | 39.05 |
| AHXN-12 | 445,541,184 | 445,509,216 | 99.99 | 0.03 | 96.64 | 91 | 38.73 |
| AHXN-13 | 401,878,944 | 401,853,600 | 99.99 | 0.03 | 96.82 | 91.48 | 38.47 |
| AHZY-1 | 401,260,320 | 401,260,320 | 100 | 0.03 | 96.09 | 89.46 | 38.74 |
| AHZY-2 | 412,822,944 | 412,791,264 | 99.99 | 0.03 | 96.06 | 89.64 | 38.45 |
| AHZY-3 | 416,973,600 | 416,939,328 | 99.99 | 0.03 | 95.44 | 87.98 | 38.82 |
| AHZY-4 | 385,636,608 | 385,636,608 | 100 | 0.03 | 95.13 | 87.19 | 39.14 |
| AHZY-5 | 437,626,944 | 437,596,992 | 99.99 | 0.03 | 96.3 | 89.94 | 38.93 |
| AHZY-6 | 457,773,120 | 457,772,832 | 100 | 0.03 | 95.58 | 88.24 | 38.91 |
| AHZY-7 | 368,606,304 | 368,606,016 | 100 | 0.04 | 93.91 | 84.44 | 39.23 |
| AHZY-8 | 438,322,752 | 438,322,176 | 100 | 0.03 | 96.12 | 89.66 | 38.3 |
| AHZY-9 | 462,105,216 | 462,070,368 | 99.99 | 0.03 | 96.3 | 89.96 | 38.52 |
| AHZY-10 | 278,766,432 | 278,748,000 | 99.99 | 0.04 | 94.48 | 86.93 | 38.07 |
| AHZY-11 | 325,957,536 | 325,957,536 | 100 | 0.03 | 94.68 | 87.1 | 38.8 |
| AHZY-12 | 364,815,072 | 364,785,984 | 99.99 | 0.04 | 94.08 | 85.95 | 39.01 |
| AHZY-13 | 334,080,288 | 334,080,288 | 100 | 0.03 | 94.79 | 87.41 | 38.2 |
| FJNP-1 | 365,080,896 | 365,061,888 | 99.99 | 0.04 | 93.94 | 85.76 | 39.45 |
| FJNP-2 | 378,006,336 | 378,006,336 | 100 | 0.03 | 94.78 | 87.42 | 39.02 |
| FJNP-3 | 334,230,336 | 334,212,480 | 99.99 | 0.04 | 94.25 | 86.34 | 39.23 |
| FJNP-4 | 324,872,064 | 324,872,064 | 100 | 0.04 | 93.11 | 83.97 | 39.49 |
| FJNP-5 | 349,291,008 | 349,271,136 | 99.99 | 0.03 | 94.7 | 87.13 | 38.48 |
| FJNP-6 | 406,645,056 | 406,645,056 | 100 | 0.03 | 94.81 | 87.48 | 38.7 |
| FJNP-7 | 307,581,984 | 307,550,592 | 99.99 | 0.03 | 95.21 | 88.25 | 38.22 |
| FJNP-8 | 320,105,664 | 320,076,288 | 99.99 | 0.03 | 94.94 | 87.58 | 38.98 |
| FJNP-9 | 360,144,000 | 360,143,136 | 100 | 0.03 | 94.9 | 87.37 | 39.12 |
| FJNP-10 | 323,923,680 | 323,921,952 | 100 | 0.03 | 95.53 | 88.75 | 38.43 |
| FJNP-11 | 367,534,656 | 367,510,752 | 99.99 | 0.03 | 95.53 | 88.84 | 38.76 |
| FJNP-12 | 290,779,488 | 290,756,736 | 99.99 | 0.03 | 95.38 | 88.54 | 38.99 |
| FJNP-13 | 368,352,000 | 368,352,000 | 100 | 0.03 | 95.03 | 87.63 | 39.49 |
| FJNP-14 | 251,956,512 | 251,954,496 | 100 | 0.03 | 96.22 | 90.19 | 39.25 |
| FJNP-15 | 357,865,920 | 357,844,032 | 99.99 | 0.03 | 95.62 | 88.97 | 39.05 |
| GXGZ-1 | 385,605,504 | 385,602,624 | 100 | 0.03 | 95.71 | 88.91 | 38.98 |
| GXGZ-2 | 426,642,624 | 426,640,032 | 100 | 0.03 | 96.28 | 90.22 | 38.37 |
| GXGZ-3 | 474,045,408 | 474,041,664 | 100 | 0.03 | 96.25 | 90.18 | 38.62 |
| GXGZ-4 | 397,446,912 | 397,444,608 | 100 | 0.03 | 96.06 | 89.65 | 39.09 |
| GXGZ-5 | 439,047,936 | 439,045,920 | 100 | 0.03 | 95.37 | 87.94 | 39.02 |
| GXGZ-6 | 426,189,888 | 426,187,008 | 100 | 0.03 | 96.88 | 91.55 | 38.84 |
| GXGZ-8 | 438,238,080 | 438,234,912 | 100 | 0.03 | 96.28 | 90.15 | 38.83 |
| GXGZ-9 | 441,220,320 | 441,219,168 | 100 | 0.03 | 96.5 | 90.77 | 38.88 |
| GXGZ-10 | 397,713,312 | 397,710,144 | 100 | 0.03 | 96.34 | 90.32 | 38.8 |
| GXGZ-11 | 524,687,328 | 524,684,448 | 100 | 0.03 | 95.93 | 89.25 | 38.12 |
| GXGZ-12 | 465,620,832 | 465,618,528 | 100 | 0.03 | 95.57 | 88.5 | 38.5 |
| GXGZ-13 | 415,163,520 | 415,125,504 | 99.99 | 0.03 | 95.59 | 88.38 | 39.16 |
| GXLZ-1 | 357,535,584 | 357,491,520 | 99.99 | 0.04 | 94.17 | 86.31 | 38.92 |
| GXLZ-2 | 377,293,248 | 377,267,328 | 99.99 | 0.04 | 93.51 | 84.93 | 39.07 |
| GXLZ-3 | 343,342,944 | 343,342,944 | 100 | 0.03 | 94.84 | 87.53 | 38.57 |
| GXLZ-4 | 308,517,408 | 308,492,928 | 99.99 | 0.03 | 95.15 | 88.28 | 38.78 |
| GXLZ-5 | 369,963,648 | 369,932,832 | 99.99 | 0.04 | 94.44 | 86.72 | 39.01 |
| GXLZ-6 | 310,806,144 | 310,805,856 | 100 | 0.04 | 94.22 | 86.25 | 39.02 |
| GXLZ-7 | 334,074,240 | 334,045,728 | 99.99 | 0.04 | 93.96 | 85.75 | 39.11 |
| GXLZ-8 | 349,372,800 | 349,372,800 | 100 | 0.04 | 93.67 | 85.25 | 39.33 |
| GXLZ-9 | 351,168,768 | 351,138,528 | 99.99 | 0.04 | 94.52 | 86.95 | 38.72 |
| GXLZ-10 | 314,458,848 | 314,432,352 | 99.99 | 0.04 | 93.98 | 85.85 | 39.16 |
| GXLZ-11 | 352,086,624 | 352,047,744 | 99.99 | 0.04 | 92.76 | 83.34 | 39.25 |
| GXLZ-12 | 331,761,600 | 331,761,312 | 100 | 0.03 | 94.52 | 86.8 | 38.77 |
| GXLZ-13 | 357,100,992 | 357,100,992 | 100 | 0.04 | 94.58 | 87.08 | 38.83 |
| GZLP-2 | 413,490,816 | 413,489,088 | 100 | 0.03 | 95.19 | 88.09 | 39.3 |
| GZLP-3 | 355,284,576 | 355,284,576 | 100 | 0.04 | 94.06 | 86 | 38.69 |
| GZLP-4 | 328,631,040 | 328,631,040 | 100 | 0.04 | 94.13 | 86.06 | 38.98 |
| GZLP-5 | 310,222,368 | 310,222,368 | 100 | 0.04 | 94.19 | 86.14 | 39.27 |
| GZLP-6 | 349,573,536 | 349,573,248 | 100 | 0.04 | 94.36 | 86.62 | 39.08 |
| GZLP-7 | 312,648,768 | 312,616,512 | 99.99 | 0.04 | 94.53 | 86.67 | 38.7 |
| GZLP-9 | 307,608,192 | 307,581,120 | 99.99 | 0.04 | 94.08 | 86 | 38.89 |
| GZLP-10 | 340,812,576 | 340,812,576 | 100 | 0.03 | 95 | 87.9 | 38.69 |
| GZLP-11 | 305,726,112 | 305,726,112 | 100 | 0.04 | 94.59 | 87.21 | 39.08 |
| GZLP-12 | 298,504,800 | 298,481,184 | 99.99 | 0.04 | 93.72 | 85.39 | 39.19 |
| GZLP-13 | 347,751,648 | 347,751,360 | 100 | 0.04 | 94 | 85.97 | 39.25 |
| GZZY-1 | 362,654,784 | 362,636,064 | 99.99 | 0.03 | 95.79 | 89.38 | 39.29 |
| GZZY-2 | 333,739,872 | 333,716,256 | 99.99 | 0.03 | 95.64 | 88.94 | 39.08 |
| GZZY-3 | 412,744,608 | 412,743,744 | 100 | 0.03 | 95.44 | 88.49 | 38.31 |
| GZZY-4 | 369,766,944 | 369,750,528 | 100 | 0.03 | 94.94 | 87.56 | 38.67 |
| GZZY-5 | 293,453,280 | 293,427,072 | 99.99 | 0.03 | 95.09 | 87.78 | 39.06 |
| GZZY-6 | 299,765,088 | 299,762,208 | 100 | 0.03 | 95.05 | 87.69 | 39.28 |
| GZZY-8 | 290,148,480 | 290,126,880 | 99.99 | 0.03 | 95.44 | 88.44 | 38.8 |
| GZZY-10 | 297,621,216 | 297,606,816 | 100 | 0.03 | 95.1 | 87.81 | 39.12 |
| GZZY-11 | 339,656,256 | 339,635,232 | 99.99 | 0.03 | 95.91 | 89.64 | 38.9 |
| GZZY-13 | 337,900,032 | 337,875,840 | 99.99 | 0.03 | 94.83 | 87.28 | 39.33 |
| HBES-1 | 309,574,944 | 309,573,216 | 100 | 0.03 | 95.14 | 87.92 | 38.85 |
| HBES-2 | 372,760,992 | 372,759,264 | 100 | 0.03 | 94.75 | 87.05 | 39.52 |
| HBES-3 | 348,887,232 | 348,858,720 | 99.99 | 0.03 | 95.3 | 88.28 | 38.47 |
| HBES-4 | 385,245,216 | 385,242,048 | 100 | 0.03 | 95.35 | 88.45 | 39.01 |
| HBES-5 | 378,445,248 | 378,443,520 | 100 | 0.03 | 95.26 | 88.27 | 39.48 |
| HBES-6 | 359,741,376 | 359,739,648 | 100 | 0.03 | 94.8 | 87.19 | 39.7 |
| HBES-7 | 300,200,832 | 300,166,848 | 99.99 | 0.03 | 96.1 | 89.94 | 39.24 |
| HBES-8 | 351,167,328 | 351,165,600 | 100 | 0.03 | 95.42 | 88.59 | 39.2 |
| HBES-9 | 328,923,648 | 328,922,208 | 100 | 0.03 | 95.5 | 88.84 | 38.85 |
| HBES-10 | 324,291,744 | 324,271,584 | 99.99 | 0.03 | 95.45 | 88.58 | 39.35 |
| HBES-11 | 353,505,600 | 353,503,008 | 100 | 0.03 | 95.09 | 87.8 | 37.57 |
| HBES-12 | 392,696,064 | 392,663,232 | 99.99 | 0.04 | 94.21 | 86.41 | 36.94 |
| HBES-13 | 359,906,688 | 359,906,688 | 100 | 0.03 | 95.91 | 89.27 | 37.86 |
| HBYS-1 | 338,905,152 | 338,867,424 | 99.99 | 0.04 | 94.25 | 86.16 | 39.44 |
| HBYS-2 | 354,563,424 | 354,562,272 | 100 | 0.03 | 95.63 | 89.02 | 38.7 |
| HBYS-3 | 327,718,944 | 327,682,080 | 99.99 | 0.03 | 95.85 | 89.64 | 38.78 |
| HBYS-4 | 361,326,816 | 361,292,544 | 99.99 | 0.03 | 95.15 | 88 | 39.2 |
| HBYS-5 | 278,206,560 | 278,182,368 | 99.99 | 0.03 | 94.94 | 87.52 | 39.03 |
| HBYS-6 | 325,084,896 | 325,083,168 | 100 | 0.03 | 94.62 | 86.88 | 39.25 |
| HBYS-7 | 343,435,968 | 343,434,528 | 100 | 0.04 | 94.48 | 86.62 | 39.97 |
| HBYS-8 | 346,974,336 | 346,946,688 | 99.99 | 0.03 | 95.28 | 88.31 | 39.07 |
| HBYS-9 | 315,674,208 | 315,650,016 | 99.99 | 0.03 | 94.6 | 86.88 | 39.37 |
| HBYS-10 | 323,994,240 | 323,992,800 | 100 | 0.04 | 93.43 | 84.38 | 39.92 |
| HBYS-11 | 306,396,000 | 306,394,848 | 100 | 0.03 | 95.27 | 88.13 | 38.95 |
| HBYS-12 | 361,077,120 | 361,073,952 | 100 | 0.03 | 95.3 | 88.4 | 39.17 |
| HBYS-13 | 301,725,216 | 301,698,432 | 99.99 | 0.03 | 95 | 87.84 | 38.49 |
| HZXH-1 | 352,444,320 | 352,444,032 | 100 | 0.03 | 94.81 | 87.52 | 38.56 |
| HZXH-2 | 327,341,664 | 327,314,880 | 99.99 | 0.03 | 94.72 | 87.32 | 39.02 |
| HZXH-3 | 321,056,064 | 321,031,872 | 99.99 | 0.04 | 94.44 | 86.61 | 39.57 |
| HZXH-4 | 279,221,184 | 279,203,040 | 99.99 | 0.03 | 95.69 | 89.12 | 39.22 |
| HZXH-5 | 378,593,856 | 378,565,920 | 99.99 | 0.03 | 94.89 | 87.65 | 39.08 |
| HZXH-6 | 374,909,472 | 374,884,416 | 99.99 | 0.03 | 95.09 | 88.06 | 39.29 |
| HZXH-7 | 347,285,664 | 347,266,944 | 99.99 | 0.03 | 94.97 | 87.69 | 38.95 |
| JXFY-1 | 343,704,672 | 343,703,232 | 100 | 0.03 | 96.09 | 89.5 | 38.78 |
| JXFY-2 | 401,479,776 | 401,479,488 | 100 | 0.03 | 95.75 | 88.78 | 38.8 |
| JXFY-3 | 296,356,320 | 296,340,768 | 99.99 | 0.03 | 96.22 | 89.96 | 38.25 |
| JXFY-4 | 298,594,080 | 298,569,600 | 99.99 | 0.03 | 96.28 | 90.09 | 38.45 |
| JXFY-5 | 357,018,048 | 356,995,584 | 99.99 | 0.03 | 96.03 | 89.43 | 38.97 |
| JXFY-6 | 272,491,776 | 272,491,776 | 100 | 0.03 | 95.46 | 87.84 | 39.42 |
| JXFY-7 | 301,428,288 | 301,428,288 | 100 | 0.03 | 96.91 | 91.41 | 38.66 |
| JXFY-8 | 283,221,504 | 283,220,928 | 100 | 0.03 | 96.24 | 89.86 | 38.85 |
| JXFY-9 | 355,584,960 | 355,560,192 | 99.99 | 0.03 | 96.5 | 90.56 | 39.06 |
| JXFY-10 | 384,224,256 | 384,223,680 | 100 | 0.03 | 96.3 | 90.03 | 38.69 |
| JXFY-11 | 422,213,472 | 422,212,320 | 100 | 0.03 | 95.82 | 88.78 | 37.77 |
| JXFY-12 | 410,389,056 | 410,389,056 | 100 | 0.03 | 95.63 | 88.45 | 38.43 |
| SHSJ-1 | 421,130,880 | 421,095,168 | 99.99 | 0.03 | 96.66 | 91.22 | 38.61 |
| SHSJ-2 | 384,010,848 | 383,977,152 | 99.99 | 0.03 | 96.81 | 91.59 | 38.59 |
| SHSJ-3 | 455,366,592 | 455,364,000 | 100 | 0.03 | 96.06 | 89.59 | 38.91 |
| SHSJ-4 | 456,074,496 | 456,072,192 | 100 | 0.03 | 96.15 | 89.96 | 38.92 |
| SHSJ-5 | 462,519,648 | 462,516,768 | 100 | 0.03 | 95.39 | 88.09 | 38.96 |
| SHSJ-6 | 465,103,872 | 465,102,144 | 100 | 0.03 | 95.06 | 87.34 | 39.09 |
| SHSJ-7 | 498,282,336 | 498,228,768 | 99.99 | 0.03 | 96.16 | 89.89 | 38.6 |
| SHSJ-8 | 437,268,960 | 437,266,944 | 100 | 0.03 | 95.43 | 88.18 | 38.74 |
| SHSJ-9 | 438,786,432 | 438,783,840 | 100 | 0.04 | 93.7 | 84.44 | 38.73 |
| SHSJ-10 | 458,195,616 | 458,192,736 | 100 | 0.03 | 96.22 | 90.09 | 38.67 |
| SHSJ-11 | 437,120,640 | 437,118,912 | 100 | 0.03 | 96.28 | 90.17 | 38.93 |
| SHSJ-12 | 324,836,640 | 324,810,432 | 99.99 | 0.03 | 95.91 | 89.44 | 37.89 |
| SHSJ-13 | 361,001,088 | 360,971,136 | 99.99 | 0.03 | 96.1 | 89.73 | 39.22 |
| ZJLQ-1 | 346,101,984 | 346,078,080 | 99.99 | 0.03 | 94.69 | 87.37 | 39.29 |
| ZJLQ-2 | 360,362,016 | 360,338,976 | 99.99 | 0.04 | 94.32 | 86.43 | 39.2 |
| ZJLQ-3 | 286,280,064 | 286,280,064 | 100 | 0.03 | 95.26 | 88.39 | 39.56 |
| ZJLQ-4 | 389,081,376 | 389,057,184 | 99.99 | 0.03 | 94.95 | 87.83 | 39.27 |
| ZJLQ-5 | 382,554,432 | 382,536,000 | 100 | 0.04 | 94.18 | 86.21 | 40.21 |
| ZJLQ-6 | 388,006,272 | 388,006,272 | 100 | 0.04 | 94.25 | 86.43 | 39.57 |
| ZJLQ-7 | 388,156,320 | 388,130,688 | 99.99 | 0.03 | 94.41 | 86.71 | 39 |
| ZJLQ-8 | 405,191,232 | 405,160,992 | 99.99 | 0.04 | 93.83 | 85.55 | 39.75 |
| ZJLQ-9 | 346,191,840 | 346,191,840 | 100 | 0.03 | 95.04 | 87.91 | 38.88 |
| ZJLQ-10 | 291,951,072 | 291,951,072 | 100 | 0.03 | 95.35 | 88.66 | 38.81 |
| ZJLQ-11 | 330,713,280 | 330,678,432 | 99.99 | 0.03 | 94.7 | 87.2 | 39.01 |
| ZJLQ-12 | 362,530,080 | 362,503,584 | 99.99 | 0.04 | 94.48 | 86.75 | 39.8 |
| ZJLQ-13 | 342,409,824 | 342,409,824 | 100 | 0.04 | 94.29 | 86.29 | 40.06 |

**Table S4.** Statistics of SNP type.

| nTs | nTv | Ts/Tv | Total |
| --- | --- | --- | --- |
| 338908 | 120656 | 2.808 | 459564 |

nTs. Number of SNPs belong to transition type; nTv. Number of SNPs belong to transvertion type; Ts/Tv. The number ratio of transition SNPs to transversion SNPs.
